# Real-world community hospital hyperglycemia management in noncritically ill, type 2 diabetic patients: a comparison between basal-bolus insulin and correctional insulin

**DOI:** 10.3389/jpps.2024.13074

**Published:** 2024-06-11

**Authors:** Caiyun J. Yang, Chelsey Bourgeois, Elina Delgado, William Graham, Melissa A. Burmeister

**Affiliations:** ^1^ Slidell Memorial Hospital, Slidell, LA, United States; ^2^ William Carey University School of Pharmacy Department of Pharmacy Practice, Biloxi, MS, United States; ^3^ William Carey University School of Pharmacy Department of Pharmaceutical Sciences, Biloxi, MS, United States

**Keywords:** basal-bolus insulin, type 2 diabetes mellitus (T2DM), inpatient glycemic management, pharmacy-led diabetes stewardship, correctional insulin

## Abstract

**Purpose:**

This study evaluated the safety and efficacy of two insulin regimens for inpatient hyperglycemia management: combination short-plus long-acting insulin (basal-bolus insulin regimen, BBIR) vs. short-acting insulin only (correctional insulin only regimen, CIOR).

**Methods:**

Chart reviews identified noncritically ill patients with pre-existing type 2 diabetes mellitus receiving insulin injections. Study participants (N = 138) were divided into BBIR (N = 104) and CIOR (N = 34) groups. Data for the entire duration of each patient’s stay were analyzed.

**Results:**

The primary outcome of percent hyperglycemic days was higher in BBIR vs. CIOR (3.97 ± 0.33% vs. 1.22 ± 0.38%). The safety outcome of percent hypoglycemic events was not different between BBIR and CIOR (0.78 ± 0.22% vs. 0.53 ± 0.37%). Regarding secondary outcomes, the percentage of euglycemic days was lower in BBIR vs. CIOR (26.74 ± 2.97% vs. 40.98 ± 5.91%). Overall blood glucose (BG) and daily insulin dose were higher in BBIR vs. CIOR (231.43 ± 5.37 vs. 195.55 ± 6.25 mg/dL and 41.36 ± 3.07 vs. 5.02 ± 0.68 units, respectively). Insulin regimen-associated differences in hyperglycemia and daily insulin dose persisted after adjusting for covariates.

**Conclusion:**

Our observations linking BBIR to worse glycemic outcomes differ from those reported in the randomized controlled Rabbit 2 and Rabbit 2 Surgery trials. This discrepancy can be partly explained by the fact that BBIR patients displayed worse glycemic baselines. Also, there was no diabetes stewardship team to monitor BG and modify insulin therapy, which is relevant since achieving euglycemia in BBIR patients requires more dose adjustments. This study highlights challenges with standard inpatient glycemic management and calls for further research assessing the benefits of pharmacist-led diabetes stewardship.

## Introduction

Type 2 diabetes mellitus (T2DM) is the most prevalent metabolic disorder in the U.S., affecting 37.2 million people (∼10% of the population) [1]. More than 25% of hospitalized patients present with diabetes as a comorbidity. These patients are at a greater risk of developing hyperglycemia while being treated for other active hospital problems. Hyperglycemia is associated with prolonged length of stay (LOS) in the hospital, increased risk of infection, higher hospital costs, and complications after discharge, including death [2, 3]. However, hyperglycemia is frequently overlooked and improperly managed in noncritically ill, hospitalized patients, which contributes to both short- and long-term complications [2, 3]. Many variables (*e.g.*, hypermetabolic stress, altered nutrient intake, impaired mobility, liver dysfunction, renal disease, etc.) can affect blood glucose (BG) levels. Multiple studies of pharmacy-led inpatient hyperglycemic management have demonstrated success in improving patient outcomes [4–8]. However, most hospitals do not have a designated team (*i.e.*, diabetes stewardship) to assess and adjust insulin regimens (*e.g.*, sliding scale intensity, dose, etc.).

Historically, hyperglycemia has been managed by a sliding scale insulin regimen, which entails bolus administration of short-acting insulin only [9]. Both the American Diabetes Association (ADA) Standard of Medical Care in Diabetes and Endocrine Society Clinical Practice guidelines have made recommendations (1) in support of the use of a basal-bolus insulin regimen (BBIR) in noncritically ill patients in the hospital setting and (2) against the sole use of correctional scale insulin (also referred to as a sliding scale insulin regimen) due to increased risk of hyperglycemia [10–12]. Specifically, Endocrine Society guidelines recommended the use of a BBIR rather than a CIOR for hyperglycemia management in noncritically ill, hospitalized patients in 2012. ADA guidelines followed suit in 2016. Changes to the guidelines were based largely on outcomes from the RABBIT 2 and RABBIT 2 Surgery trials [13, 14]. Both trials were randomized, multi-center, and open-label studies that assessed hyperglycemia management in noncritically-ill, hospitalized patients with T2DM. Each trial reported that, compared to sliding scale insulin therapy, a BBIR is associated with a lower incidence of hyperglycemia and no significant difference in severe hypoglycemic events [13, 14]. Despite these recommendations, there were patients who received a correctional insulin only regimen (CIOR) at Slidell Memorial Hospital (SMH). This is partly due to provider concern regarding the occurrence of hypoglycemia with a BBIR due to reduced nutritional intake in the hospital, as well as thoughts that hyperglycemia may be associated with acute medical or surgical illness. The objective of this study was to compare the safety and efficacy of inpatient hyperglycemia management with BBIR vs. CIOR in T2DM patients in a 230-bed community hospital with no established diabetic stewardship.

## Methods

This was a single-center, retrospective, Ochsner IRB-approved study that included patients who were admitted to noncritical units at Slidell Memorial Hospital (SMH, Ochsner Health System) for at least 3 days and who were 18 years of age and older, had a T2DM diagnosis, and received insulin treatments. Patients who were pregnant; had a LOS longer than 21 days; received oral anti-hyperglycemic medications, parenteral nutritional, or systemic steroids; or required dialysis were excluded. Data were collected from SMH’s electronic medical record between 1 March 2022 and 31 August 2022 using ICD-10 codes to identify patients who met the inclusion criteria. Patients were divided into two study groups based on hyperglycemic control approach. Patients in the BBIR group received (1) long-acting insulin detemir or insulin glargine plus short-acting insulin (insulin aspart or insulin regular) or (2) neutral protamine Hagedorn (NPH) plus insulin aspart. Patients in the CIOR group received insulin aspart or insulin regular (SQ) alone. All insulin agents were administered subcutaneously. In both groups, short-acting insulin aspart and insulin regular doses were based on point-of-care (POC) testing (POCT) BG readings, which were taken before meals and at bedtime, as well as a standard sliding scale insulin protocol. The insulin sliding scale has three intensities: low, medium, and high. Medium intensity was selected for most patients. Low intensity was selected for patients with end-stage renal disease, end-stage liver disease, a body mass index (BMI) of <25, or a high risk for hypoglycemia. High intensity was not typically selected initially. SMH nursing staff administered the insulin dose according to the BG reading by the preselected insulin sensitivity scale. Of note, NPH was seldom prescribed in this study, and insulin regular was typically changed to insulin aspart after one dose. The primary treatment goal was to achieve euglycemia while preventing hypoglycemia, with the following BG targets: prandial BG of 70–140 mg/dL and random BG of 140–180 mg/dL. Hypoglycemia was defined as a BG reading of less than 70 mg/dL. To change treatment intensity, the provider was required to discontinue the existing order and submit a new order set. Per SMH’s hypoglycemia protocol, all insulin orders included standing orders of 50% dextrose injection, oral glucose gel, and glucagon as well as nursing orders to perform POCT. POCT BG readings were repeated when BG was less than 50 mg/dL or greater than 400 mg/dL. Protocol required that providers be notified if a subsequent BG reading repeatedly measured less than 50 mg/dL or greater than 400 mg/dL.

Data for the entire duration of each patient’s stay were collected and analyzed. For instances when baseline data were missing (*i.e.*, for serum creatinine, A1C, hemoglobin, and number of patients receiving a diabetic diet), available data were analyzed and the corresponding N adjusted accordingly. Primary endpoints 1 and 2 were the percentage of days during the entire LOS that a patient’s daily average BG was greater than 180 or 240 mg/dL, respectively. BG values were obtained via both POCT and lab metabolic panels, with the greater than 240 mg/dL outcome assessed to mimic the RABBIT 2 trial. The primary safety endpoint was the percentage of events during the entire LOS that a patient’s BG reading was less than 70 mg/dL. The secondary objectives were to measure the percentage of days during the entire LOS that a patient’s daily average BG was in the euglycemic range (*i.e.*, 70–180 mg/dL), overall BG, daily dose of insulin received, average LOS, and percentage of patients who received a diabetic diet. Overall as well as subgroup data are presented. Specifically, the groups were dichotomized into two age categories: (1) younger than 65 years of age and (2) 65 years of age and older. This delineation allowed for observations to be compared in the context of non-elderly vs. elderly populations.

Statistical analyses were performed using GraphPad Prism (v. 8.3.1) and IBM SPSS Statistical (v. 29.0.2.0) software. For categorical variables, a Fisher’s exact test was performed to determine differences between groups. For continuous variables, a student’s t-test was performed; data are presented as mean ± standard error of the mean (SEM). Standard deviation (SD) is also provided for all endpoint data. Two-way multivariate analysis of variance (2-W MANOVA), linear regression, multivariate analysis of covariance (MANCOVA), and Quade’s nonparametric analysis of covariance (ANCOVA) were performed to determine main and interactive effects of insulin regimen and age category on study outcomes as well as to adjust for differences in baseline characteristics between groups that could potentially impact study outcomes and confound data interpretation. Statistical significance was set to *p* < 0.05.

## Results

**FIGURE 1:**
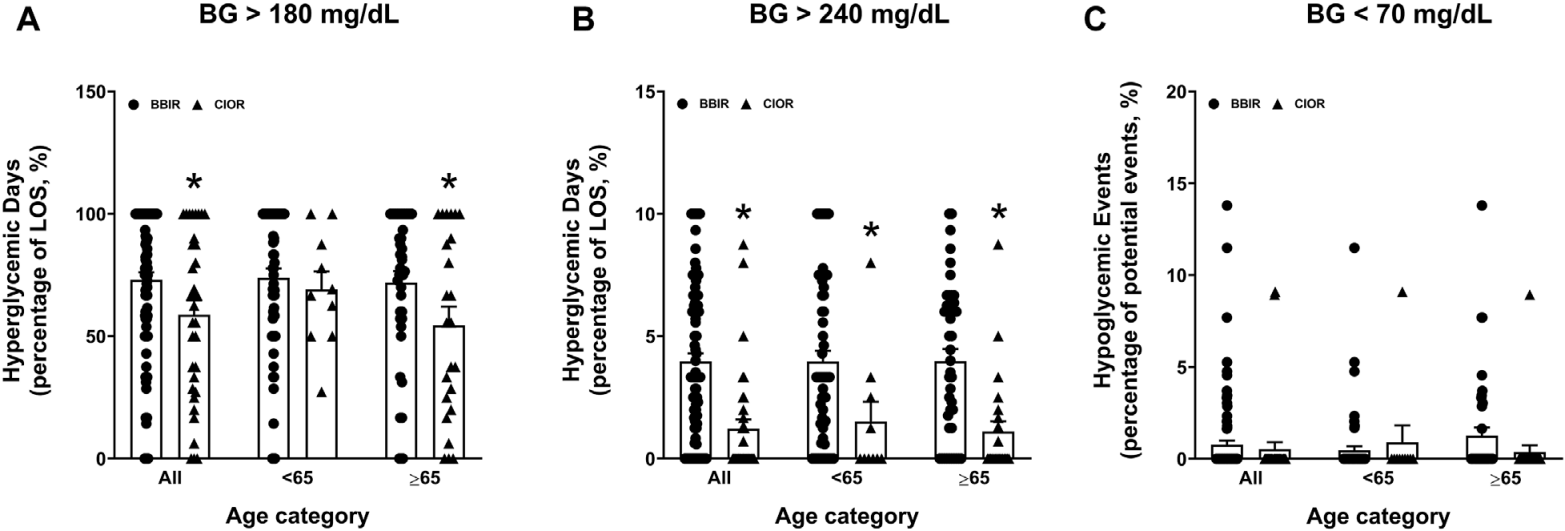
Primary and safety endpoint data in basal-bolus insulin regimen (BBIR)- vs. correctional insulin only regimen (CIOR)-treated patients. Data represent the **(A)** percentage of hyperglycemic days with average BG greater than 180 mg/dL (primary endpoint 1), **(B)** percentage of hyperglycemic days with average BG greater than 240 mg/dL (primary endpoint 2), and **(C)** percentage of hypoglycemic events with average BG less than 70 mg/dL (safety endpoint) over the course of each patient’s entire length of stay in the BBIR vs. CIOR group. Data are presented for all ages as well as for younger than 65 years of age (<65) and 65 years of age and older (≥65) subgroups. Averaged data represent mean ± SEM. *p < 0.05 vs. BBIR.

**FIGURE 2:**
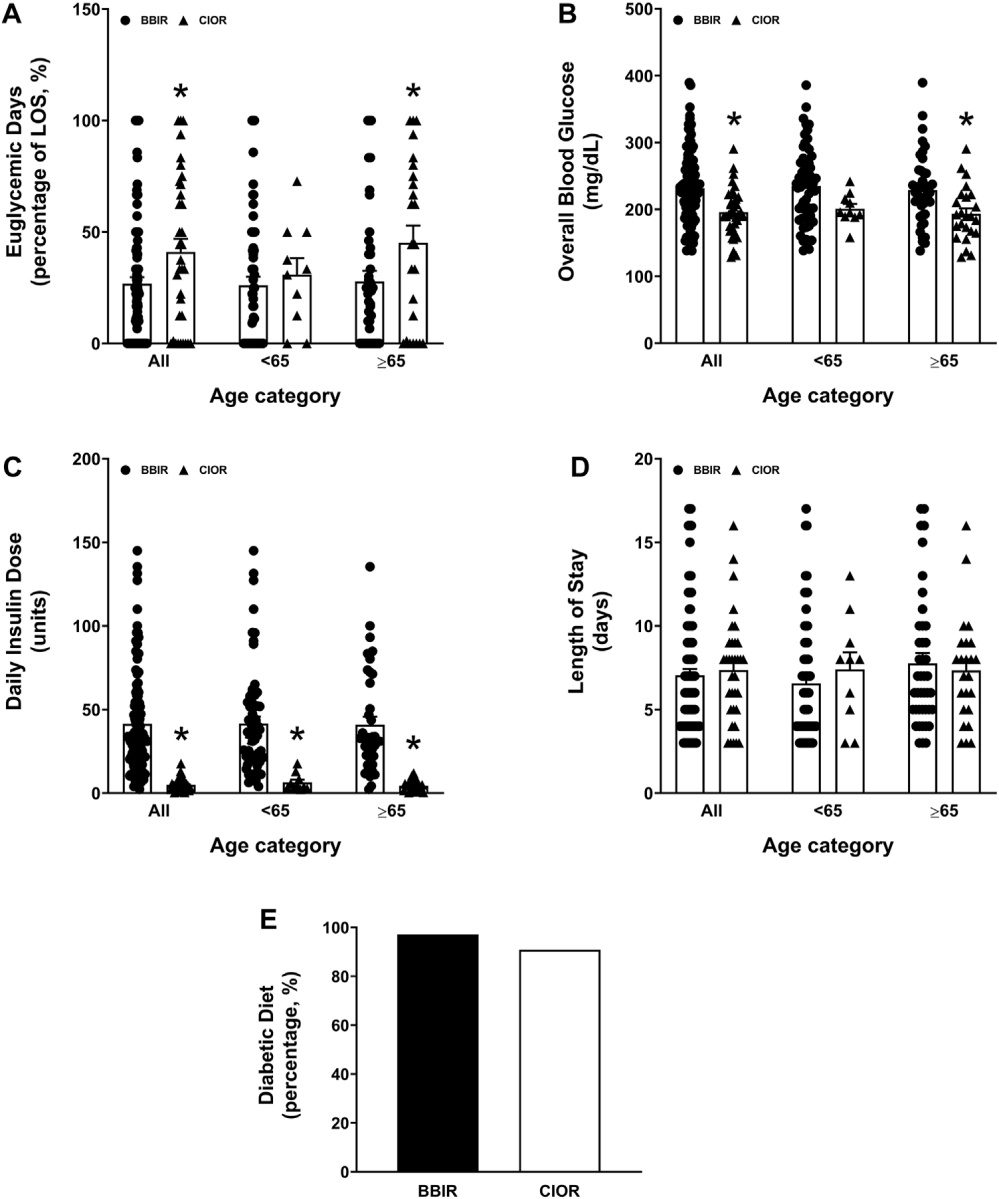
Secondary endpoints of percentage of euglycemic days, daily insulin dose, overall blood glucose, length of hospital stay, and whether or not patients were placed on a diabetic diet. Data represent the **(A)** percentage of euglycemic days with average BG between 70 and 180 mg/dL over the course of each patient’s entire length of stay, **(B)** overall blood glucose, **(C)** daily insulin dose, **(D)** length of stay, and **(E)** percentage of patients who received a diabetic diet. Data are presented for all ages as well as for younger than 65 years of age (<65) and 65 years of age and older (≥65) subgroups. Averaged data represent mean ± SEM. *p < 0.05 vs. BBIR.

Data were collected from a total of 150 patients. Eleven patients were excluded due to the use of basal insulin only. One additional patient was excluded due to a LOS greater than 3 weeks. Therefore, a total of 138 patients were included in the study, with 104 in the BBIR group and 34 in the CIOR group. Baseline characteristics are presented in Table 1. Age is presented as an overall average as well as an average for patients either younger than 65 years of age or 65 years of age and older. Of note, first FBG upon admission was 222.07 ± 8.95 mg/dL in the BBR group vs. 159.82 ± 8.38 mg/dL in the CIOR group. Specifically, first FBG measurements were frequently in the hyperglycemic range (BG > 140; BBIR: 82.69%; CIOR: 67.65%), with few readings in the hypoglycemic range (BG < 70) BBIR: 1.92%; CIOR: 2.94%). These differences in first FBG were observed in both age categories. Primary endpoint 1 was 73.06 ± 2.97% in BBIR patients vs. 58.76 ± 5.86% in CIOR patients, which was significantly lower overall as well as in the ≥65 years of age group (Figure 1A). Primary endpoint 2 was 3.97 ± 0.33% in BBIR patients vs. 1.22 ± 0.38% in CIOR patients, which was significantly lower overall as well as across both age categories (Figure 1B). The primary safety endpoint was 0.78 ± 0.22% in BBIR patients vs. 0.53 ± 0.37% in CIOR patients, which was not significantly different overall or in either age category (Figure 1C). Regarding secondary endpoints, the percentage of days that daily average BG was in the euglycemic range was 26.74 ± 2.97% in the BBIR group and 40.98 ± 5.91% in the CIOR group, which was significantly higher overall as well as in the ≥65 years of age group (Figure 2A). Overall BG was significantly higher in the BBIR vs. CIOR group (231.43 ± 5.37 vs. 195.55 ± 6.25 mg/dL), both overall as well as in the ≥65 years of age group (Figure 2B). Daily insulin dose was also significantly higher in the BBIR vs. CIOR group (41.36 ± 3.07 vs. 5.02 ± 0.68 units, respectively), both overall as well as across both age categories (Figure 2C). LOS was not significantly different between the BBIR vs. CIOR group (7.05 ± 0.37 vs. 7.35 ± 0.55 days, respectively), both overall or in either age category (Figure 2D). The majority of patients in both groups received a diabetic diet (BBIR group: 97.12%; CIOR group: 90.91%) (Figure 2E). These endpoint data are also presented in Table 2.

**TABLE 1:** Baseline characteristics.

Variable		BBIR (N)	CIOR (N)
Age, years	All	60.27 ± 1.39 (104)	71.50 ± 2.29 (34)
	<65	50.86 ± 1.21 (62)	55.36 ± 2.23 (10)
	≥65	74.15 ± 0.97 (42)	78.22 ± 1.79 (24)
Male gender, percentage		56.73 (104)	55.88 9 (34)
Race, percentage	African American	16.35 (17)	20.59 (7)
	Asian	0.96 (1)	0.00 (0)
	American Indian or Alaska Native	0.96 (1)	2.94 (1)
	White	81.73 (85)	76.47 (26)
Weight, kg		92.70 ± 2.57 (104)	84.23 ± 3.83 (34)
Height, cm		171.80 ± 1.24 (104)	169.72 ± 1.97 (34)
BMI, kg/m^2^		31.18 ± 0.86 (104)	29.82 ± 1.47 (34)
Serum creatine, mg/dL		1.77 ± 0.19 (99)	1.74 ± 0.27 (30)
First fasting blood glucose, mg/dL	All ages	222.07 ± 8.95 (104)	159.82 ± 8.38 (34)
	Age <65	244.87 ± 11.84 (62)	167.60 ± 22.82 (10)
	Age ≥65	188.40 ± 11.97 (42)	156.58 ± 7.51 (24)
A1C, percentage	All ages	9.60 ± 0.25 (92)	6.95 ± 0.22 (30)
	Age <65	10.23 ± 0.32 (59)	7.14 ± 0.36 (8)
	Age ≥65	8.46 ± 0.33 (33)	6.88 ± 0.27 (22)
Hgb, g/dL		11.49 ± 0.29 (65)	11.00 ± 0.46 (13)

**TABLE 2:** Primary, safety, and secondary endpoint data.

Endpoint data	Age category	BBIR (N = 104)	CIOR (N = 34)	*p*-value
Primary endpoints				
Hyperglycemic days, BG greater than 180 mg/dL, percentage±SEM (SD)	All	73.06 ± 2.97 (30.33)	58.76 ± 5.86 (34.16)	0.0223^*^
	<65	73.87 ± 3.85 (30.34)	69.10 ± 7.34 (23.22)	0.6366
	≥65	71.86 ± 4.73 (30.66)	54.45 ± 7.63 (37.39)^*^	0.0447^*^
Hyperglycemic days, BG greater than 240 mg/dL, percentage±SEM (SD)	All	3.97 ± 0.33 (3.32)	1.22 ± 0.38 (2.21)	<0.0001^*^
	<65	3.96 ± 0.44 (3.46)	1.51 ± 0.82 (2.59)	0.0354^*^
	≥65	3.98 ± 0.49 (3.15)	1.10 ± 0.43 (2.09)	0.0002^*^
Primary safety endpoint				
Hypoglycemic events, BG less than 70 mg/dL, percentage±SEM (SD)	All	0.78 ± 0.22 (2.25)	0.53 ± 0.37 (2.15)	0.5710
	<65	0.44 ± 0.22 (1.72)	0.91 ± 0.91 (2.87)	0.4973
	≥65	1.28 ± 0.43 (2.80)	0.37 ± 0.37 (1.82)	0.1728
Secondary endpoints				
Euglycemic days, BG between 70 and 180 mg/dL, percentage±SEM (SD)	All	26.74 ± 2.97 (30.29)	40.98 ± 5.91 (34.44)	0.0230^*^
	<65	26.03 ± 3.85 (30.33)	30.91 ± 7.34 (23.22)	0.6294
	≥65	27.80 ± 4.72 (30.57)	45.18 ± 7.72 (37.80)	0.0458^*^
Overall blood glucose, mg/dL, average ± SEM (SD)	All	231.43 ± 5.37 (54.77)	195.55 ± 6.25 (36.45)	0.0005^*^
	<65	234.80 ± 7.22 (56.88)	201.05 ± 7.29 (23.05)	0.0695
	≥65	228.66 ± 8.16 (52.85)	193.26 ± 8.37 (40.99)	0.0062^*^
Daily insulin dose, units, average ± SEM (SD)	All	41.36 ± 3.07 (31.34)	5.02 ± 0.68 (3.97)^*^	<0.0001
	<65	41.61 ± 4.09 (32.23)	6.50 ± 1.61 (5.10)^*^	0.0011^*^
	≥65	40.99 ± 4.68 (30.35)	4.41 ± 0.68 (3.32)^*^	<0.0001^*^
Length of stay, days, average ± SEM (SD)	All	7.05 ± 0.37 (3.79)	7.35 ± 0.55 (3.18)	0.6733
	<65	6.56 ± 0.46 (3.62)	7.40 ± 1.02 (3.24)	0.4944
	≥65	7.76 ± 0.61 (3.97)	7.33 ± 0.66 (3.23)	0.6543
Number of patients receiving a diabetic diet/total number of patients (percentage)		101/104 (97.12)	30/33 (90.91)	

SD: standard deviation; SEM: standard error of the mean; *p < 0.05.

Statistical analyses of outcomes data are presented in Supplementary Table S1. Two-way MANOVA determined that the observed differences in the percentage of hyperglycemic (BG greater than 240 mg/dL) days, overall BG, and daily insulin dose between the BBIR and CIOR groups across both age categories were significantly associated with insulin regimen. Furthermore, there was no association between age category and any of the study outcomes, nor were any study outcomes influenced by an interaction between age category and insulin regimen. Linear regression confirmed that age category (*i.e.*, younger vs. older) did not affect patient outcomes. First FBG, baseline A1C, and age category were identified as potential covariates that could influence study endpoints. Bivariate correlation analysis revealed that first FBG was positively correlated with A1C and negatively correlated with age; furthermore, age was negatively correlated with A1C. All of these correlations were statistically significant. To avoid collinearity, first FBG was selected as a covariate for the parametric MANCOVA model. Although the assumption of homogeneity of variance-covariance was met, that of normal distribution (with the exception of overall BG) was not, so MANCOVA results could not be reliably interpreted. Alternatively, Quade’s nonparametric ANCOVA was performed. The association between CIOR and a reduced percentage of hyperglycemic (BG greater than 240 mg/dL) days and daily insulin dose remained significant after adjusting for baseline differences in age category and first FBG. Adjusting for covariates also revealed a significant association between insulin regimen and the percentage of hypoglycemic events.

## Discussion

Although the RABBIT 2 and RABBIT 2 Surgery trials were rigorous and well-designed, they were small in size, with only 130 and 375 study participants, respectively. They were conducted in an ideal environment, whereby daily insulin monitoring and dose adjustments were performed by skilled research teams. In most hospitals, however, there are no designated diabetes management teams. Furthermore, many variables such as liver failure, kidney failure, and response to insulin can influence a physician’s choice of insulin therapy and the subsequent outcomes. Moreover, most noncritically ill, hospitalized patients are not admitted due to hyperglycemia but rather because of another acute illnesses. Hyperglycemia management is, thus, oftentimes not the prioritized intervention during hospitalization.

Here, study results were analyzed collectively across all ages as well as according to age. The two age categories of (1) younger than 65 years of age and (2) 65 years of age and older were selected to assess the effect of elderly age on study outcomes. Several of our study results differ from those previously reported in the RABBIT 2 and RABBIT Surgery trials. Namely, a lower percentage of hyperglycemic days was observed in the CIOR group. This effect of the CIOR was significant only in elderly study participants at the BG of greater than 180 mg/dL endpoint, whereas it was significant in both non-elderly and elderly patients at the BG of greater than 280 mg/dL endpoint. The percentage of hypoglycemic events was similar across both insulin regimens and age categories. A higher percentage of euglycemic days was observed in the CIOR group, which was significant only in elderly study participants. However, these differences in glycemic outcomes were not associated with age category or an interactive effect of age category and insulin regimen. Thus, results based on overall age best summarize our study findings. BBIR patients were admitted with a higher mean first FBG than CIOR patients, who, in turn, were older than BBIR patients. The effect of CIOR to reduce overall BG during an entire hospital LOS was significant only in elderly patients. When differences in baseline first FBG and age category were adjusted, the percentage of days when daily BG was greater than 240 mg/dL and daily insulin dose remained significantly higher in the BBIR group, indicating that these observations were, in fact, associated with insulin regimen. In line with the other glycemic outcomes, a significant effect of CIOR to reduce the percentage of hypoglycemic events was also observed when controlling for covariates.

In contrast to our findings, the RABBIT 2 and RABBIT Surgery trials reported that hospitalized patients in the BBIR group had a higher percentage of measurements in the euglycemic range. Furthermore, the ADA and Endocrine Society Clinical Practice guidelines recommend the use of BBIR rather than CIOR because it maintains patients in the euglycemic range for a greater percentage of time. Nevertheless, our observations corroborate findings from a large retrospective study that associated BBIR with fewer euglycemic days than CIOR [15]. Similar to findings reported in the RABBIT 2 study, a higher average total daily insulin dose was used in the BBIR vs. CIOR group [14]. In the RABBIT 2 study, the average total daily insulin dose was 42 units in the basal-bolus group vs. 12 units in the sliding scale insulin group, with an average A1C of 8.8% in both groups [14]. Similar differences in the average total daily insulin doses in the BBIR and CIOR groups were observed in the present study. However, average A1C values differed between the BBIR vs. CIOR groups (9.60% ± 0.25 vs. 6.95% ± 0.22, respectively). Of note, these A1C values correlate with BG values that track with the respective first FBG measurements of the two groups. This is one reason why A1C and first FBG were not both selected as covariates, and first FBG was chosen since some A1C data were missing. These data suggest that BG control was not prioritized in either group, which is consistent with the notion that hyperglycemia is generally overlooked and poorly managed in noncritically ill, hospitalized patients. The guideline recommendation to replace the ineffective CIOR with BBIR for inpatient hyperglycemia management has been advocated for nearly two decades. However, inpatient glycemic control is highly complex, as it is influenced by many factors, requiring daily assessments, frequent dose adjustments, and regimen switching when necessary. Hence, the need for diabetic stewardship.

The study is limited by its retrospective design and differences in sample size and baseline characteristics. For instance, patients in the BBIR group (N = 104) were younger than those in the CIOR group (N = 34) (60.27 ± 1.39 vs. 71.50 ± 2.29 years of age, respectively). Higher BMI, first FBG, and A1C values in the BBIR vs. CIOR group indicate that the diabetes in BBIR patients was not as well controlled prior to hospitalization. Therefore, achieving euglycemia in BBIR patients is likely to require more frequent insulin dose adjustments compared to what is needed in CIOR patients. Indeed, the average daily insulin dose administered to BBIR patients was substantially greater. Poor glycemic control prior to admission, coupled with consistent hyperglycemia during hospitalization, could lead to additional complications in BBIR patients. Hospital chart records were not assessed for reason(s) for admission, active acute illnesses, other comorbidities, percentage of meal intake, total carbohydrate intake per meal, or time of meal following administration of insulin, all of which could affect BG levels. However, nearly all patients were placed on a diabetic diet. Furthermore, protocol compliance was not assessed. Namely, whether patients received an appropriate dose of insulin based on POCT BG readings per SMH’s protocol was not determined. Whether an appropriate sliding scale insulin dose (*i.e.*, low, moderate, or high) for achieving euglycemia was selected or adjusted upon failure to achieve euglycemia were not determined. Whether basal insulin dose was adjusted when FBG levels were consistently elevated was also not determined. Diabetic treatments prior to admission, mortality, and readmission rates were also not assessed. Lastly, lack of generalizability is a significant limitation, as the study was conducted at only a single institution.

When selecting an appropriate insulin regimen to optimally manage hyperglycemia in T2DM patients, it is best to choose one that is individual-specific (*i.e.*, based on A1C value, fasting vs. prandial BG levels, age, body weight, presence of comorbidities, and at-home insulin dose prior to admission when applicable). BBIR, CIOR, and, ultimately, the insulin dose administered should be based on individual POCT BG measurements and inpatient carbohydrate intake. It is sometimes necessary to switch between insulin treatment regimens and frequently adjust the insulin sliding scale intensity to achieve euglycemia. This study reports that patients with uncontrolled T2DM require insulin dose adjustments more frequently. In a diabetes stewardship program, pharmacists are actively involved in glycemic management, including daily BG assessment and insulin dose adjustment. Through close monitoring of BG and adjustment of insulin dose on an as-needed basis, diabetic stewardship can improve patient outcomes and increase the use of guideline-recommended insulin regimens [7]. Pharmacist-led diabetes management may also lead to increased physician satisfaction with glycemic management, allowing them to focus on treating the acute active illness. Diabetes stewardship programs have been recognized by multiple organizations, and their establishment can facilitate successful Joint Commission accreditation as an Advanced Inpatient Diabetes Care center [16]. Moving forward, initiating diabetic stewardship that entails the collaborative efforts of physicians, pharmacists, and nurses to optimally adjust insulin therapy may expedite achieving euglycemia. If a stationed unit pharmacist is available to review BG readings and assist with daily insulin dose adjustments, then perhaps euglycemia could be achieved sooner. Here we report that, although 77% of patients were prescribed guideline-recommended treatment, BG levels remained high, suggesting that insulin regimens may not always be selected/adjusted appropriately. Future studies comparing the success rate of glycemic control with or without diabetic stewardship would be informative.

## Data Availability

The original contributions presented in the study are included in the article/Supplementary Material, further inquiries can be directed to the corresponding author.
