# Supplementary material for: Real-world community hospital hyperglycemia management in noncritically ill, type 2 diabetic patients: a comparison between basal-bolus insulin and correctional insulin
Source: J Pharm Pharm Sci. 2024 Jun 11;27:13074. doi: 10.3389/jpps.2024.13074 (PMC11196384; doi:10.3389/jpps.2024.13074)
Supplement: Supplementary file 1 [file Table1.docx]

**Supplemental Table 1. Statistical analyses of main, interactive, and covariate effects.**

| **STATISTICAL TEST** | **COVARIATES** | **INDEPDNENT VARIABLE(S)** | **DEPENDENT VARIABLE** | **MAIN**  **AND INTERACTIVE EFFECTS** | **X̅** | **PARAMETER ESTIMATES** | | **p-value** |
| --- | --- | --- | --- | --- | --- | --- | --- | --- |
|  |  |  |  |  |  | **95% Confidence interval** | |  |
|  |  |  |  |  |  | **Lower bound** | **Upper bound** |  |
| **2-W MANOVA** | **None** | **Insulin regimen,**  age category | Hyperglycemic days, BG greater than 180 mg/dL | **Insulin regimen** | **17.142** | **1.547** | **33.277** | **0.099** |
|  |  |  |  | **Age category** | **14.646** | **-8.690** | **37.982** | **0.215** |
|  |  |  |  | **Interaction** | **-12.638** | **-39.059** | **13.784** | **0.346** |
|  |  |  | Hyperglycemic days, BG greater than 240 mg/dL | **Insulin regimen** | **2.879** | **1.304** | **4.453** | **<0.001^*^** |
|  |  |  |  | **Age category** | **0.406** | **-1.910** | **2.721** | **0.770** |
|  |  |  |  | **Interaction** | **-0.423** | **-3.045** | **2.200** | **0.750** |
|  |  |  | Hypoglycemic events, BG less than 70 mg/dL | **Insulin regimen** | **0.902** | **-0.216** | **2.019** | **0.634** |
|  |  |  |  | **Age category** | **0.514** | **-1.130** | **2.158** | **0.730** |
|  |  |  |  | **Interaction** | **-1.354** | **-3.216** | **0.508** | **0.153** |
|  |  |  | Euglycemic days, BG between 70 and 180 mg/dL | **Insulin regimen** | **-17.382** | **-33.274** | **-1.489** | **0.099** |
|  |  |  |  | **Age category** | **-14.275** | **-37.652** | **9.102** | **0.233** |
|  |  |  |  | **Interaction** | **12.507** | **-13.961** | **38.975** | **0.352** |
|  |  |  | **Overall blood glucose** | **Insulin regimen** | **35.398** | **9.339** | **61.456** | **0.002^*^** |
|  |  |  |  | **Age category** | **7.795** | **-30.536** | **46.125** | **0.526** |
|  |  |  |  | **Interaction** | **-1.649** | **-45.047** | **41.750** | **0.940** |
|  |  |  | **Daily insulin dose** | **Insulin regimen** | **36.585** | **22.647** | **50.522** | **<0.001^*^** |
|  |  |  |  | **Age category** | **2.098** | **-18.404** | **22.599** | **0.817** |
|  |  |  |  | **Interaction** | **-1.478** | **-24.690** | **21.734** | **0.900** |
| **Liner regression** | **None** | **Age category** | Hyperglycemic days, BG greater than 180 mg/dL | **BBIR** | **-2.008** | **-14.085** | **10.069** | **0.742** |
|  |  |  |  | **CIOR** | **-14.646** | **-40.716** | **11.424** | **0.261** |
|  |  |  | Hyperglycemic days, BG greater than 240 mg/dL | **BBIR** | **0.017** | **-1.306** | **1.340** | **0.980** |
|  |  |  |  | **CIOR** | **-0.406** | **-2.121** | **1.310** | **0.634** |
|  |  |  | Hypoglycemic events, BG less than 70 mg/dL | **BBIR** | **0.840** | **-0.040** | **1.720** | **0.061^*^** |
|  |  |  |  | **CIOR** | **-0.514** | **-2.179** | **1.151** | **0.534** |
|  |  |  | Euglycemic days, BG between 70 and 180 mg/dL | **BBIR** | **1.769** | **-10.292** | **13.830** | **0.772** |
|  |  |  |  | **CIOR** | **14.276** | **-12.043** | **40.594** | **0.277** |
|  |  |  | **Overall blood glucose** | **BBIR** | **-6.147** | **-28.064** | **15.771** | **0.579** |
|  |  |  |  | **CIOR** | **-7.795** | **-36.034** | **20.445** | **0.578** |
|  |  |  | **Daily insulin dose** | **BBIR** | **-0.619** | **-13.101** | **11.863** | **0.922** |
|  |  |  |  | **CIOR** | **-2.097** | **-5.090** | **0.896** | **0.163** |
| **Quade’s nonparametric ANCOVA** | **First FBG,**  **age category** | **Insulin regimen** | Hyperglycemic days,  BG greater than 180 mg/dL | **Insulin regimen** | **1.938** | **-10.916** | **14.792** | **0.766** |
|  |  |  | Hyperglycemic days,  BG greater than 240 mg/dL | **Insulin regimen** | **20.493** | **7.784** | **33.201** | **0.002^*^** |
|  |  |  | Hypoglycemic events,  BG less than 70 mg/dL | **Insulin regimen** | **10.428** | **1.365** | **19.491** | **0.024^*^** |
|  |  |  | Euglycemic days,  BG between 70 and 180 mg/dL | **Insulin regimen** | **-1.917** | **-14.691** | **10.858** | **0.767** |
|  |  |  | **Overall blood glucose** | **Insulin regimen** | **11.606** | **-1.120** | **24.332** | **0.074** |
|  |  |  | **Daily insulin dose** | **Insulin regimen** | **49.936** | **38.595** | **61.277** | **<0.001^*^** |
| ^*^p < 0.05. | | | | | | | | |
